# Provision for Epileptics1Read before the National Conference of Charities and Correction, held at Nashville, Tenn., May 23-28, 1894.

**Published:** 1894-08

**Authors:** William Pryor Letchworth

**Affiliations:** Commissioner of the New York State Board of Charities


### PROVISION FOR EPILEPTICS.[1]

By WM. PRYOR LETCHWORTH, LL. D.,
Commissioner of the New York State Board of Charities.

Various theories as to the cause of epilepsy have been advanced by those who have studied the subject scientifically, but none of them have stood the test of general application; and at the present time the most learned in the study of the subject frankly admit that the nature of the disease is still a mystery. Dr. Ludwig Hirt, in his noted work on Diseases of the Nervous System, says: "The structure as well as the physiological functions of the human brain are, up to the present time, so little understood that we are far from having any sure basis upon which to lay the foundations of a cerebral pathology." He further says: "By epilepsy in the stricter sense of the term we designate a functional neurosis, the seat of which is still unknown."

Dr. Jules Christian, physician to the National Hospital at Charenton, France, says: "Nobody really doubts that epilepsy is a disease of the brain ; upon that all are agreed, but no one has been able to determine with any precision the part of the organ affected, nor the nature of the lesion which produces the disease. All the researches of pathological anatomy have hitherto been at variance." After discussing different theories on the subject, beginning with that of Dr. Marshall Hall, he says: "To anyone asking my opinion as to the approximate cause of epilepsy, I should simply reply, I do not know."

Though physicians differ as to the nature of epilepsy, and the wisest admit that they have not yet been able to fathom its mysteries, it is an established fact that certain medical and moral treatment will render less frequent the terrible manifestations of the disease, and in some instances prevent their recurrence. It is, therefore, of the greatest importance that such provision be made for this unfortunate class as seems best suited to their needs. The fact that epilepsy is not thoroughly understood, instead of discouraging research into its mysteries, argues strongly in favor of turning upon the subject the concentrated light of scientific investigation and study.

The epileptic is in one sense an outcast. In his own home his presence is distressful and disturbing ; the privileges of the church and of places of amusement are denied him ; it is difficult for him

1. Read before the National Conference of Charities and Correction, held at Nashville, Tenn., May 23-28, 1894.

to find employment; and eventually, the progress of his malady having been gradually accelerated by his unhappy environment, he finds his way into the almshouse or the insane asylum, neither of which is a suitable place for him. In the almshouse he cannot receive the care and treatment which his peculiar infirmity requires, and his presence in the insane asylum is often injurious to the insane, especially the convalescing; moreover, a sense of the injustice of enforced confinement and association with lunatics, precipitates a crisis in his own mental condition.

While it is generally admitted by those who have studied this disease with reference to the best interests of those afflicted by it and of society, that care and treatment different from that provided for any other class of dependents is necessary for them, it is, nevertheless, true that very limited special provision has been made for epileptics in comparison with their numbers. According to returns made to the New York State Board of Charities, there were 614 epileptics in the poorhouses and almshouses of the State of New York, September 30, 1893. It is estimated that there are 12,000 in the State, and 120,000 in the United States. These numbers are based on an estimate of about two to every one thousand of the population. In Germany the number is estimated to be at least one to every one thousand of the population. A recent census taken of epileptics in the canton of Arrau, Switzerland, made the ratio 2.42 to every one thousand of the population. These estimates include epilepsy of all types. It is thought that the ratio of this class in England is not less than it is in the German states.

The principal homes for epileptics are in Germany, and most of those established within recent years were founded upon the colony plan, which is now regarded as the most advanced system of care for this class that has yet been devised. In a late report of a special committee of the Charity Organization Society of London, which was appointed to make a scientific inquiry into the public and charitable provision for the care and training of epileptics and feeble-minded, deformed and crippled persons, we have some valuable suggestions respecting the proper provision for epileptics. The large number of eminent physicians and experienced charity workers engaged in this inquiry entitles the following conclusion reached by them to careful consideration:

For all alike, for the furtherance of self-control and for healthy enjoyment, a well-ordered home life is required, These things—school education, employment of the most suitable and varied kinds, and homelife—the colony system provides. As house after house is built for the settlers, the classification becomes more and more complete for all purposes. Each house should be in its internal administration a separate unit, under the charge of a home superintendent or house father. There is thus always large scope for expansion according to actual demand. A large staff of nurses is necessary, and for these special provision must be made. Medically, if the serious nature of the disease be taken into account, the colony system, with careful medical treatment, produces the best results. For the worst cases, and to provide against the constant ailments of many of the colonists, hospital accommodation is necessary ; and, for the study of the disease, the fullest opportunity must be given to scientific research and treatment.

Dr. Peterson,[1] who, while First Assistant Physician at the Hudson River State Hospital, had a large number of epileptics under his charge, and who has studied their needs by personal observations at Bielefeld and elsewhere, thus remarks on the kind of care necessary for this class :

There is but one kind of institution which can meet the case of those who suffer from this disease. No asylum, no large hospital, no single vast building in a great city, is appropriate for the purpose. It must be an establishment combining many unusual features. It must have schools and teachers for the education of the young epileptics ; it must have offices, shops of all kinds, stores, dairy, farm, gardens, granaries, for as they grow up, these patients should acquire trades or professions ; it must have a group of small hospital and asylum buildings where such as are sick or mentally infirm may be cared for ; it must have skilled physicians ; it must have a church, a theatre, a gymnasium, and a bathing establishment ; it must have, finally, a pathological laboratory presided over by the keenest pathologist obtainable, so that in the course of time a cause and a cure may be discovered for this terrible disease. Such a place would not be a hospital in the ordinary sense of the term ; it would be a village in itself, a colony for epileptics.

It may be of interest to glance briefly at the origin of the colony system. In doing so we must turn our attention to Europe. Some time prior to 1848, John Bost, pastor of a Protestant church at La Force, near Bordeaux in France, out of his sympathy for friendless girls, set out to build a home for them adjoining his church. The members of his congregation, who were poor, aided him with the labor of their hands, and he begged means while preaching in France, England and Switzerland to secure his object.

1. Dr. Frederick Peterson, Attending Physician to the New York Hospital for Nervous Diseases ; Pathologist to the New York City Insane Asylums ; Chief-of-Clinic, Nervous Department, Vanderbilt Clinic, College of Physicians and Surgeons, New York.

He succeeded in opening his home in 1848. But other classes of the unfortunate likewise appealing to his sympathies, he soon built another house for young girls who were suffering from incurable diseases, or who were feeble-minded, or who were blind or becoming so. He next put up a house for epileptic girls, and, later, one for epileptic women, following with others for those who were dependent and afflicted with disease. His last house was built in 1881. The whole formed a group of families who lived as nearly as possible like families in ordinary homes. Pastor Bost believed in the efficacy of outdoor life in the country as well as medicine, in healing certain diseases, in the advantages of working in the fields and garden, of caring for animals, and in the contemplation of the works of Nature. From these homes for epileptics, which form a part of the mixed colony founded by John Bost, at La Force, came the first practical suggestion of colonizing epileptics and caring for them in family groups. The method thus originated formed the basis of a system which has been followed to a greater or less extent on the continent of Europe where provision has been made for this class.

The celebrated colony of Bielefeld, in Westphalia, was organized upwards of twenty-five years ago, through the humane efforts of Pastor von Bodelschwingh, a Lutheran clergyman, under the auspices of the Provincial Committee of the Inner Mission in Rhineland and Westphalia. It is a private charitable institution, largely supported by receipts from private patients and voluntary subscriptions, but partially by payments from public authorities. It has among its beneficiaries 1,100 epileptics, separately classified, who are under the gentle ministrations of the Westphalian Brotherhood and the Westphalian Deaconesses. This colony, with its plain, unpretentious buildings distributed over 1,350 acres of land, its church, school, gymnasium, places for entertainment, hospital, carpenter shop, blacksmith shop, shoe shop, saddlery, tailor shop, basket weaver's establishment, iron foundry, mill, seed assorting house, book bindery, printing establishment, bakery, brick kiln, farm buildings for the care of all kinds of stock, cultivated fields, meadows, orchards, gardens, groves, embellished grounds, trellised vines and attractive dwellings, has more the characteristics of home, family and natural life than any other existing institution for epileptics. It has been so well described by Dr. Peterson, who visited it in 1886; also in the report made upon it to the Charity Organization Society of London, by Miss E. Burdon Sanderson, who visited it in 1892, and in a recently published work of Julie Sutter, entitled A Colony of Mercy, that further reference to it seems unnecessary.

There are but two or three homes for epileptics in England, and these are of quite recent origin. The Maghull Home was founded in 1889 mainly through the efforts of Dr. William Alexander and the benefactions of Mr. Cox, a wealthy resident of Liverpool. The principal building of the home is an old manor house, which is situated in the midst of neat lawns and gardens surrounded by shaded meadows, about seven miles from the city of Liverpool. The cases admitted are chiefly those in which there is a reasonable prospect of cure or amelioration. The system of treatment includes a well-ordered home-life, plain diet, careful supervision and employment. This small work, undertaken as an experiment, has been so successful that an attempt is being made to extend it on a larger scale in other parts of England.

At Godalming, in Surrey, the Countess of Meath has lately established a home for epileptic women and girls. It has a capacity for fifty patients. The work was inspired by a visit made by this estimable lady to Bielefeld, where she was deeply impressed with the wisdom and benevolence of the work conducted there by Pastor von Bodelschwingh.

There are two or three hospitals for the paralyzed and epileptic in London, but the largest and most important of these, the National Hospital, does not admit chronic cases, and the other two treat the ordinary epileptic only as an out-patient. The majority of the charitable institutions refuse admission to epileptics ; and all charitable workers, whether medical or lay, have found it next to impossible to obtain employment for those who suffer from fits, with the result that the workhouse, poor-law infirmaries and lunatic asylums become the only places where these unfortunate people can be received.

The National Society for the Employment of Epileptics is now making an energetic effort to benefit this large class of sufferers, by establishing homes where eight hundred or more sane epileptics may be provided with suitable employment under proper supervision. With this aim in view, the society has just purchased a farm in Buckinghamshire, near the Village of Chalfont, St. Peters, and commenced improvements upon it. The plan proposed is :

To provide a home for those necessitous epileptics who are able and willing to work, but for whom their friends are unable to procure employment on account of the affliction which bars their admission into ordinary fields of industry. It is intended that the cottages shall be arranged for these, and shall each accommodate, according to their size, from ten to twenty epileptics. The sexes will be separated, as will also the children from the adults. Market gardening, spade and barrow labor, cow-keeping, dairy work and poultry farming will be the first industries ; then gardening and fruit culture, and later on will follow bootmaking, carpentering book-binding, printing and other industries ; and for the women, laundry work, sewing, cooking and various domestic services.

In this praiseworthy movement, from which much good is anticipated, some of the most prominent men and women in England are engaged.

To Ohio belongs the honor of being the first State in the Union to provide a State institution exclusively for epileptics. It was through the forcible presentation to the legislature, by the State Board of Charities, of the neglect, sufferings and needs of this peculiar class that the Governor of the State, in 1890, was authorized by the legislature to appoint a commission to select a site for an institution for their special care and medical treatment. This action, with subsequent legislation, resulted in the establishment of the Asylum for Epileptics and Epileptic Insane at Gallipolis, the corner-stone of which was laid November 12, 1891. For this new departure in our country, on behalf of a much neglected class, we are largely indebted to the persistent efforts of General R. Brinkerhoff, President of the Ohio State Board of Charities.

It unfortunately happened in this case, as it frequently does in founding State institutions, that a narrow policy stood in the way of a right beginning. In order to utilize a property already belonging to the State, and formerly used for another purpose, a site was selected having an insufficiency of land. Thus the planting of an institution on the colony or village plan became, to a large degree impracticable. The whole estate comprises only 107 acres. The buildings form a single group, and, like many other of our State institutions, are designed more to create an imposing effect than to attain the naturalness and diversity of home or family life. The buildings are on the pavilion or cottage plan, in the midst of which stands the administration building. Five cottages have been completed, with accommodation for 250 men ; and it is expected that four more will be ready this Summer, for the accommodation of 200 women. The plan embraces twelve cottages for fifty patients each. Those erected are of gray sandstone, fire-proof, two stories high, and built at a cost of $15,000 each. In the further erection of cottages it has been decided to place them at a greater distance from the main group, in order to conform more nearly to the colony system. The aim is to secure the means of classification for those differently affected,—hospital treatment for those requiring it, and education and useful employment for those who may be benefited thereby.

In Massachusetts there was opened, in 1882, the Hospital Cottages for Children, at Baldwinvsille. The institution was first organized as a private charity, but is now governed by five trustees appointed by the Governor, and fourteen appointed by the corporation. It has been liberally aided by the State. Children under fourteen years of age are admitted who are suffering from epileptic or epileptiform seizures; children suffering from nervous disorders, not feeble-minded ; children with deformities, diseases of the joints and infantile paralysis ; also those needing surgical operations and fitting supports. On September 30, 1893, the hospital contained 103 children. The whole number treated during the fifteen previous months was 170, about two-thirds of whom were epileptics.

In 1892, the Governor of Massachusetts sent to the legislature a message respecting the making of State provision for epileptics, upon which he recommended early and favorable action. His message was accompanied by a report made to him by a committee of experts of the Massachusetts Medical Society, advocating the establishment of an institution in the form of cottage hospitals for epileptics. This report the Governor had previously submitted to the State Board of Lunacy and Charity, which had approved it. The legislature gave the subject some consideration, but referred it to the next General Court. No action was taken during the session of 1893. The Board of Lunacy and Charity, in alluding to this subject in their report dated January, 1894, use the following language :

> The matter is one that demands prompt attention. The number of these unfortunates is constantly increasing, and, while almost every other class of the sick, the poor and the afflicted are provided for, no special arrangement is made for adult epileptics, and their only refuge seems to be the insane hospital, in whose crowded wards they are wholly out of place, or the town almshouse, where their only prospect is increased suffering and gradual decay.

In Pennsylvania, at the Training School for Feeble-Minded, at Elwyn, two buildings, one for boys and the other for girls, are now set apart for epileptic children.

A hospital for the treatment of sane epileptics has just been organized, upon a limited scale, in Philadelphia, in connection with St. Clement Church parish. The building occupied was formerly used and known as St. Clement's Hospital.

The Lunacy Committee of the Board of Public Charities of Pennsylvania, in their report for 1893, state that 575 epileptics are detained under the lunacy law in the various institutions for the insane in that State. The committee make a strong plea for a State institution for the exclusive accommodation, care and treatment, upon an industrial basis, for this class, the wretched condition of the majority of which, it is asserted, demands relief from the legislature.

Through the instrumentality of Dr. A. E. Osborne, Superintendent of the California Home for the Care and Training of Feeble-Minded Children, at Santa Clara, this institution secured legislation, in 1887, permitting it to establish a department for epileptics, which it subsequently did by erecting a separate cottage building, capable of accommodating seventy-five children ; but this is taxed beyond its capacity, there being 100 of this class now in the institution.

Admissions are restricted to those showing marked mental enfeeblement. Dr. Osborne writes, under date of May 9, 1894 : "It is our intention, after the completion of our main building, to add other small buildings or cottages to our epileptic department, that we may extend the benefits of the institution to the more curable and hopeful class of epileptics."

A law was recently passed by the Michigan legislature providing for the care and treatment of epileptics and the feeble-minded in separate buildings on the cottage plan. A farm has been purchased to carry out this project and a contract made for putting up buildings.

A new custodial building for women and girls has just been opened at the School for the Feeble-Minded at Faribault, in Minnesota, and a department for epileptics, with special supervisory care, has been established. Mr. Hart, Secretary of the Board of Corrections and Charities of that State, says : "We have already, I think, under public care about 120 epileptics. There is some sentiment in the State in favor of a separate institution for epileptics, and I think it possible that our Board may recommend to the next legislature the creation of such an institution."

In Maryland the plan of buying a small country place and beginning an epileptic colony is being carried out by the King's Daughters of Baltimore.

In some other states, state boards of charities and charity organization societies have long been, and are still, endeavoring to secure more humane and intelligent care for epileptics. In New York State the attention of the legislature was directed to the necessity for some special provision for this class by the State Commissioner in Lunacy in his first report, as far back as 1873; and in his subsequent reports Dr. Ordronaux repeatedly emphasized the importance of the subject.

The State Board of Charities, in its report for the year 1878, directed the attention of the legislature to the lack of proper provision for this class and appealed for State intervention in their behalf.

The present State Lunacy Commission, in its report for 1891, strongly advocated the establishment of a State hospital for the special care and treatment of sane epileptics.

No person in the State of New York, nor in this country, has done more to enlighten the public and influence legislation on this important question than Dr. Peterson. This he has done through addresses before medical and other societies, State conventions of superintendents of the poor, and able contributions to the public press.

Under the auspices of the State Charities Aid Association, a bill was introduced in the New York legislature in 1892, and passed the same year, directing the State Board of Charities to select a suitable site on which to establish an institution on the colony plan for the medical treatment, care, education and employment of epileptics, and to prepare plans and estimates of cost of buildings, and submit the same to the next legislature. A committee of the board, composed of its president and two commissioners, spent a considerable part of the following Summer in looking up a site, and finally recommended the purchase of a tract of land situated in the famous Genesee Valley, the garden of the State. The site was, in aboriginal days, an Indian village, and bore the Indian name of Sonyea, with the poetical signification of sunshine. The tract contains 1,872 acres of highly productive land. It was owned by the United Society of Christian Believers, commonly designated

Shakers, who, in consequence of a reduction in their numbers, desired to sell their estate and consolidate the settlement with another branch of their society. Over the property was distributed a large number of buildings, including comfortable dwellings, capacious barns, stables, workshops, and a mill having a good water-power supplied by an unfailing, quick-flowing stream, which centrally traverses and drains the whole property. Here are extensive orchards of apple, pear, peach, plum and apricot trees, with large berry and vegetable gardens, producing every variety of garden products. The place is easily accessible. A north and south railway, with a station upon the property, intersects all the east and west trunk lines of railway that pass through the State. The property is in every way admirably adapted for the purpose of an epileptic colony, it having been used and developed by the Shakers as a colony.

Based upon the report of the State Board of Charities, a bill was introduced in the legislature in 1893, by the request of the State Charities Aid Association, to purchase the Shaker property, and to establish thereon a colony for epileptics to be known as the Sonyea Colony. The bill was almost unanimously endorsed by the legislature and by the public press, but for economic reasons was not approved by the Governor. Thus, after much labor and sacrifice of time, those interested in charity work, especially in the epileptic class, were sadly disappointed.

The same bill, however, with slight modifications, was introduced under the same auspices in the legislature of 1894 by Hon. Hamilton Fish, and has become a law of the State, this act, memorable in the history of New York State charities, was approved by Governor Flower, April 26, 1894, having passed the assembly by a vote of ninety-six to four and the senate by an unanimous vote. It is designated An Act to establish an Epileptic Colony and making an appropriation therefor. For the purpose of doing away with the suggestion of an institution for a diseased class, the word epileptic was omitted from the legal name of the corporation. With a view to memorialize fittingly the distinguished public services of Hon. Oscar Craig, late President of the State Board of Charities, whose death occurred January 2, 1894, the following declaration was made in the first section of the act:

There shall be established in Livingston County, in this State, a colony for epileptics, to be known as the Craig Colony; thus named in honor of the late Oscar Craig, of Rochester, N. Y., whose efficient and gratuitous public services in behalf of epileptics and other dependent unfortunates the State desires to commemorate.

The objects of the colony are stated to be "to secure the humane, curative, scientific and economical treatment and care of epileptics, exclusive of insane epileptics."

The act requires that the buildings and improvements upon the property shall be utilized, and that a general plan shall be adopted in accord with the recommendations in the report of the State Board of Charities to the legislature in 1893, and that subsequently all building shall be made to subserve such design and recommendations and true economy.

The colony is subject to the supervision of the State Board of Charities, and the board is required to report annually what appropriations are necessary for it. The board of managers consists of five persons appointed by the Governor, by and with the advice and consent of the senate, for terms of five years, their terms so arranged that one member retires annually. They receive no compensation for their services, but are allowed their reasonable traveling and official expenses. The board of managers appoints the superintendent and treasurer, and is charged with the entire government, discipline, management and business of the colony. The superintendent is the chief executive officer, subject to the supervision and control of the board of managers, and it is required that he must be a well-educated physician, a graduate of a legally chartered medical college, with an experience of at least five years in actual practice in his profession, including one year's actual experience in a general hospital, and that he shall be certified as qualified by the civil service commission, after a competitive examination. The superintendent appoints the steward, matron and subordinate officers and teachers, and determines their salaries, subject to the approval of the State comptroller. He may, in his discretion, suspend or discharge any of his subordinates.

Epileptics of all ages residing in the State, who are dependent, are received and gratuitously supported. They are designated as State patients. Such other epileptics as can be conveniently accommodated are admitted by special agreement upon such terms as are deemed just by the superintendent, and are designated as private patients. In the reception of patients, preference is given to poor or indigent epileptics or the children of poor or indigent persons. Preference is also given to those who are partially indigent over those who are able to furnish support. For State patients there must be paid annually by the authorities sending them the sum of $30 each for clothing. Should an epileptic State patient become insaue, he is transferred to the State hospital of the district, or institution for the insane in the district, of which he was a resident prior to his admission.

Before deciding upon the selection of the Sonyea site, every possible precaution was taken by the committee of the State Board of Charities to guard against mistakes. Statistics were gathered showing the healthfulness of the locality ; a chemical analysis was made of the water, to determine its purity and suitableness for family use; a civil engineer was engaged to examine and report on the sufficiency of the water supply and the facilities for disposing of sewage ; a survey was made of the land, to determine its boundaries and acreage ; and the numerous buildings upon the property were examined by the architect,[1] and their capacity and adaptability to colony purposes considered.

The plans submitted to the legislature were based upon principles previously enunciated by Dr. Peterson, who is considered the highest authority in our land upon this subject. It is gratifying to state that the Governor has appointed him a member of the managing board and that he has since been elected its president.

It would seem, with this magnificent estate for a foundation, and the favorable auspices under which it begins its existence, that we may reasonably expect in the Craig Colony the attainment of an ideal institution.

The colony system for the care of epileptics, which has proved so beneficent on the continent of Europe, was developed there under the auspices of private and unofficial charity. Whether we shall attain as satisfactory results under a State and official system as under one directed by a spirit of pure benevolence is a question, However this may be, to organize the work in the State of New York on a large scale on the basis of private charity has been found impracticable. We may indulge the hope that the work, as undertaken by the State, will be more comprehensive than it would have been otherwise, and that the aggregate of good, if measured by the number benefited, will be greater.

---

SALINES operate in three or four hours. Croton oil in one or two hours. Jalap, gamboge and senna, in three or four hours. Rhubarb and castor oil in from four to six hours. Aloes and mandrake in from ten to fourteen hours.—*Louisville Medical Monthly.*

1. George J. Metzger, of Buffalo.

